# Fabrication of P and N Co-Doped Carbon Dots for Fe^3+^ Detection in Serum and Lysosomal Tracking in Living Cells

**DOI:** 10.3390/bios13020230

**Published:** 2023-02-05

**Authors:** Yanzhi Xing, Mei Yang, Xuwei Chen

**Affiliations:** 1 Department of Chemistry, College of Sciences, Northeastern University, Shenyang 110819, China; 2 School of Chemistry and Chemical Engineering, Liaoning Normal University, Dalian 116029, China

**Keywords:** P and N co-doped carbon dots (PNCDs), fluorescence probe, Fe^3+^ detection, lysosomal tracking

## Abstract

Doping with heteroatoms allows the retention of the general characteristics of carbon dots while allowing their physicochemical and photochemical properties to be effectively modulated. In this work, we report the preparation of ultrastable P and N co-doped carbon dots (PNCDs) that can be used for the highly selective detection of Fe^3+^ and the tracking of lysosomes in living cells. Fluorescent PNCDs were facilely prepared via a hydrothermal treatment of ethylenediamine and phytic acid, and they exhibited a high quantum yield of 22.0%. The strong coordination interaction between the phosphorus groups of PNCDs and Fe^3+^ rendered them efficient probes for use in selective Fe^3+^ detection, with a detection limit of 0.39 μM, and we demonstrated their practicability by accurately detecting the Fe^3+^ contents in bio-samples. At the same time, PNCDs exhibited high lysosomal location specificity in different cell lines due to surface lipophilic amino groups, and real-time tracking of the lysosome morphology in HeLa cells was achieved. The present work suggests that the fabrication of heteroatom-doped CDs might be an effective strategy to provide promising tools for cytology, such as organelle tracking.

## 1. Introduction

Lysosomes are acidic subcellular organelles with pH values of 4.0 to 6.0 that occur extensively in all eukaryotic cells [1,2]. As part of the cellular digestive system, lysosomes contain a variety of hydrolases and secretory proteins and constitute the terminal degradation chambers of animal cells [3,4]. They receive foreign species and degrade macromolecules to produce small-molecule nutrients, in addition to playing major parts in various physiological processes (e.g., intracellular transport, energy homeostasis, macromolecule circulation, apoptosis, autophagy metabolism, and immunologic defense). Lysosomal dysfunction is usually related to various pathologies, including inflammation, cancer, neurodegenerative diseases, and atherosclerosis [5,6,7,8,9,10]. Up to now, tremendous efforts have been expended in monitoring lysosomal activities, such as through lysosomal membrane stability tests [11], immunohistochemical (Ih) assays of lysosomal marker enzymes [12], and neutral red retention time assays [13]. The fluorescence imaging of lysosomes has recently been gaining increasing attention, as it provides a means for direct visual observation. Most fluorescent lysosome-targeting probes used in such applications are modified with weakly basic morpholine for accumulation in this acidic organelle following protonation [14]. Due to their leakage from lysosomes, these fluorescence probes might cause the pH of lysosomes to increase after long-term accumulation, leading to the risk of significantly decreased fluorescence intensity of fluorophores, making it difficult to achieve real-time monitoring of lysosome activities. Therefore, the design and development of ultrastable probes that are able to monitor the status and changes of lysosomes in real time is urgently desired for use in advancing our understanding of lysosome dynamics and functions in biological processes.

Ferric ion (Fe^3+^) is involved in cell metabolism and enzymatic catalysis and is an oxygen carrier in hemoglobin [15,16,17]. Fe^3+^ overload or paucity can disturb homeostasis in cells and leads to iron deficiency anemia (IDA), arthritis, mental decline, heart failure, diabetes, cancer, and other various diseases [18,19,20,21]. Therefore, the detection of Fe^3+^ is very important for the early recognition and diagnosis of these diseases [22]. However, the detection selectivity of most spectral probes for sensing Fe^3+^ is usually inadequate [23,24]. Therefore, the development of fluorescent probes with high specificity for Fe^3+^ is necessary.

In recent years, carbon-based nanomaterials have stood out as new nanosensors due to their unique chemical and physical properties [25,26,27]. In the family of carbon-based nanomaterials, carbon dots (CDs) have been considered dependable fluorescence probes [28,29] and have gained great attention in various fields such as biomedicine, optoelectronic devices, catalysis, sensing, and bio-imaging [30,31,32,33,34,35]. Theoretically, the physicochemical features and optical properties of CDs could be extensively tunable by modulating precursors and/or manipulating the reaction conditions in the preparation of CDs [36]. Surface passivation, surface modification, and heteroatom doping have been shown to be effective for modulating the characteristics of CDs [37,38]. Huang's group designed and synthesized CDs passivated with polyetherimide (PEI), which significantly improved the fluorescence (FL) quantum yield of CDs and the FL emission stability under different excitation energies [39]. Chen et al. synthesized CDs modified with amine groups and lauryl amine via an 1-ethyl-3-(3-dimethylaminopropyl)carbodiimide hydrochloride (EDC) coupling reaction with citric acid monohydrate and urea as carbon sources. Alkyl chains with different lengths varied the amino to carboxyl group ratio, offering CDs with different pH responses, endocytosis pathways, and organelle-targeting abilities [40]. However, the passivation and modification process was usually tedious, and most examples involved the use of toxic organic reagents. In this respect, heteroatom doping has been gaining increasing attention due to the facile synthesis and remarkable modified effects. Until now, B [41], N [42], P [43], S [44], and halogens [45] have been integrated into CDs, and the generated heteroatom-doped products have exhibited outstanding optical performances in targeted applications. For example, Manioudakis’s group revealed that the quantum yield of CDs could be improved from less than 1% to nearly 60% by increasing the amount of doped nitrogen [46]. Li et al. used a graphite rod as a carbon source to obtain P-doped CDs via the electrolysis of sodium phytate based on the ease of cleaving the C–P bond in CDs using 2,2-diphenyl-1-picrylhydrazyl (DPPH). Therefore, P-doped CDs exhibit an attractive capacity for scavenging free radicals [47]. Moreover, N and P co-doped CDs are gaining increasing popularity in different fields due to their attractive photoluminescence prosperity (Table S1).

**Scheme 1:**
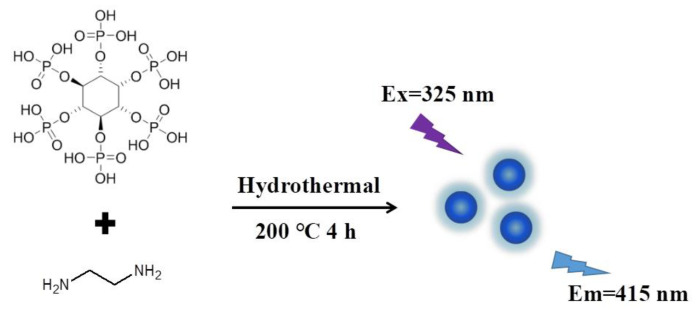
Schematic diagram for preparing fluorescent P and N co-doped CDs (PNCDs).

In this study, P and N co-doped CDs (PNCDs) were facilely prepared via a mild hydrothermal procedure using phytic acid and ethylenediamine as precursors (Scheme 1). The as-prepared PNCDs exhibited excellent photoluminescence (PL) properties, such as high quantum yield (22.0%), good photostability, and favorable resistance to variations in pH and ionic strength. PNCDs were proven to be sensitive fluorescence probes for highly selective Fe^3+^ sensing due to the specific coordination interaction between the phosphorus groups of PNCDs and Fe^3+^, and they were successfully applied in the accurate detection of Fe^3+^ contents in human serum and cells. At the same time, PNCDs showed high lysosomal location specificity in different cell lines due to their surface lipophilic amines, and the dynamic tracking of lysosomal status during the apoptosis of HeLa cells was achieved.

## 2. Materials and Methods

### 2.1. Materials

Phytic acid was purchased from McLean Biochemical Technology Co., Ltd. (Shanghai, China). Ethylenediamine, NaCl, FeCl_3_·6H_2_O, CaCl_2_, FeCl_2_, MgCl_2_·6H_2_O, HgCl_2_, MnSO_4_·H_2_O, CuSO_4_·5H_2_O, ZnCl_2_, ammonium acetate, potassium permanganate, and potassium dichromate were provided by the Damao Chemical Reagent Plant (Tianjin, China). All amino acids were purchased from Mellon Biotechnology Co., Ltd. (Dalian, China). 3-(4,5-Dimethylthiazole)-2,5-phenyltetrazolium bromide (MTT) and dimethyl sulfoxide (DMSO) were purchased from Sigma (Shanghai, China). ER-Tracker Red, Lyso-Tracker Red, and Mito-Tracker Deep Red were obtained from Beyotime Biotechnology Co., Ltd. (Shanghai, China). Cells were provided by Procell Life Science & Technology Co., Ltd. (Wuhan, China). Deionized water (18 MΩ cm) was used in all experiments.

### 2.2. Instruments and Measurements

Transmission electron microscopy (TEM) was performed using a JEM-ARM200F field-emission electron microscope (JEOL, Tokyo, Japan). Fourier transform infrared spectrum (FT-IR) were conducted using a Tensor II FT-IR spectrometer (Bruker, Saarbrucken, Germany). An X-ray photoelectron spectroscopy (XPS) analysis was recorded using an ESCALAB 250Xi analysis system (Thermo Fisher Scientific, Waltham, MA, USA). Ultraviolet-visible (UV–vis) spectra were measured using a U-3900 spectrophotometer (Hitachi, Tokyo, Japan). Fluorescent spectra were recorded using an F-7000 fluorescence spectrometer (Hitachi, Tokyo, Japan) with 1 cm quartz cells. A cytotoxicity assay was conducted using a Synergy H1 ELISA plate reader at an absorbance of 570 nm, and cell images were captured using a widefield fluorescence microscope (Olympus, Tokyo, Japan).

### 2.3. Preparation of PNCDs

Ethylenediamine (2.1 mL) and 1 mL of phytic acid (70%, *v/v*) were dissolved into 25 mL of deionized water and stirred for 15 min. Then, the mixture was sealed in a 50 mL Teflon autoclave. After heating at 200 °C for 4 h, the resultant product was collected and dialyzed using a dialysis membrane (2000 Da) to remove unreacted small molecules. The obtained solution was dried and re-dispersed into deionized water.

For comparison, N-doped carbon dots (NCDs) were also prepared using only ethylenediamine as a precursor via the same preparation procedure, and the characterizations are summarized in Figure S1.

### 2.4. PL Assay of Fe^3+^ with PNCDs

Assays of Fe^3+^ were conducted using PNCDs as the fluorescence probes. Briefly, 0.25 mL of an Fe^3+^ solution (0–1000 μM) was mixed with 50 μL of a PNCD solution (50 μg mL^−1^, prepared in PBS buffer of pH 7.40). The resultant mixture was incubated for 5 min at 25 °C, and the PL spectra were then obtained at λex = 325 nm.

The calibration curve was deduced based on the relationship between the Fe^3+^ content and the fluorescence quenching efficiency ((F_0_ − F)/F_0_, where F_0_ and F indicate the PL intensities of PNCDs in the absence/presence, respectively, of Fe^3+^).

The selectivity was investigated by adding other interfering substances (including various ions, amino acids, and proteins) into the assay system. Each measurement was implemented in triplicate to evaluate the experimental error.

### 2.5. Detection of Fe^3+^ Content in Human Serum and SH-SY5Y Cells

Whole-blood samples were obtained from Northeastern University Hospital. First, fresh human serum was mixed with an equal volume of anhydrous ethanol, followed by heating at 90 °C for 10 min. After cooling to 25°C, the serum was treated with ultrasound for 10 min and centrifuged at 5000 rpm for 5 min, and the protein sediment was discarded. Thereafter, the supernatant was carefully collected for a subsequent quantitative assay following the above PL assay procedure.

Around 70% of adherent SH-SY5Y cells were digested with pancreatin. After subsequent washing with PBS buffer (pH 7.40), deposits of cells were transferred into lysis buffer on ice for 30 min with agitation. Then, the cell solution was centrifuged at 10,000 rpm for 10 min to remove cell fragments. Finally, the supernatant was collected and diluted twice for a subsequent quantitative assay following the above PL assay procedure.

### 2.6. Cytotoxicity Assay

The standard MTT test was adopted to assess the cytotoxicity of PNCDs. HeLa cells were cultured in a 96-well plate with different concentrations of PNCDs (100 μL, prepared in a serum-free DMEM medium). After incubation for 24 h, the old DMEM medium was aspirated, and 10 μL of MTT (5 mg mL^−1^) was then added before incubation for 4 h. Next, 100 μL of DMSO was added, and the absorbance at 570 nm was recorded to determine the cell viability of each sample.

### 2.7. Subcellular Localization of PNCDs

HeLa cells were adhered to the culture dish and preincubated with 0.20 mg mL^−1^ PNCDs for 40 min before a subsequent incubation with Lyso-Tracker Red, ER-Tracker Red, or Mito-Tracker Deep Red, according to the manufacturer’s instructions. Fluorescence images were obtained using a wide-field fluorescence microscope with a 60 × objective lens. The following microscopy settings were used: blue/red channel and Ex = 330–380 nm/510–560 nm. Pearson’s correlation coefficients were analyzed using Image J software.

### 2.8. Lysosomal Tracking

HeLa cells were pretreated with 0.20 mg mL^−1^ PNCDs for 40 min, and dexamethasone (50 mM) was then added to induce cell apoptosis. After thoroughly washing with PBS, fluorescence imaging of PNCDs in the HeLa cells was performed using a wide-field fluorescence microscope. The blue fluorescence channel observation conditions were set as follows: Ex = 330–380 nm.

## 3. Results and Discussion

### 3.1. Preparation and Characterization of PNCDs

**Figure 1:**
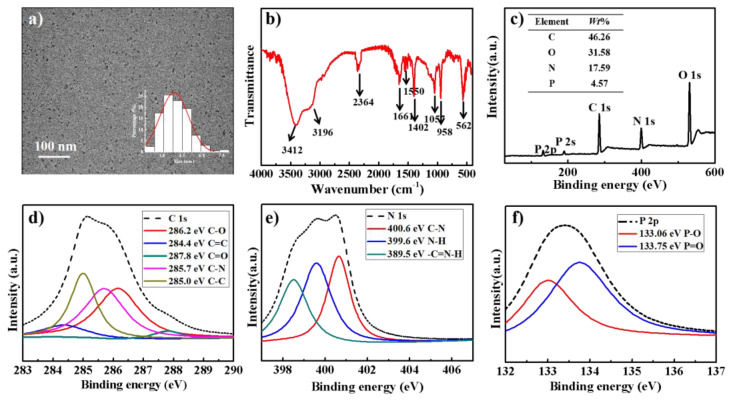
(**a**) Transmission electron microscopy (TEM) image, (**b**) Fourier transform infrared spectrum (FT-IR) (**c**) X-ray photoelectron spectroscopy (XPS) spectrum, high-resolution (**d**) C 1s, (**e**) N 1s, and (**f**) P 2p spectra of PNCDs.

Herein, PNCDs were fabricated via a simple hydrothermal route using phytic acid and ethylenediamine as precursors. To obtain highly fluorescent CDs, the impact of the mole ratio of phytic acid/ethylenediamine was studied, and PNCDs prepared with a phytic acid/ethylenediamine mole ratio of 1:20 exhibited the highest quantum yield (Table S2). This phenomenon may be due to the modulation of the electronic and chemical properties of CDs based on the proportion of co-doped N and P [48]. Figure 1a shows a TEM image of the as-prepared PNCDs. On the basis of about 100 random particles, the size distribution of PNCDs was found to range from 1.87 to 7.89 nm, with an average diameter of about 4.03 nm. The XRD pattern of PNCDs displayed one broad peak centered at 20° (Figure S2), which was attributed to the amorphous structure of the PNCDs. In the FT-IR spectra of PNCDs (Figure 1b), the band at 562 cm^−1^ represented P–O. The bands at 958 and 1057 cm^−1^ suggested the existence of P–OH and P=O, respectively. The band at 2364 cm^−1^ was ascribed to C–N vibration. The typical broad bands at 1550 and 1661 cm^−1^ were, respectively, assigned to C=N and C=O stretching vibrations. The bands at 3196 and 3412 cm^−1^, respectively, represented N–H stretching vibrations and the O–H stretching mode of the carboxylic functional group. The FT-IR spectra confirmed the presence of carboxyl, hydroxyl, amino, and phosphate groups in the obtained PNCDs. XPS was further used to study the surface components and the elemental chemical state of PNCDs. As shown in Figure 1c, the P 2s, P 2p, C 1s, N 1s, and O 1s signals appeared at 190.7, 131.0, 285.3, 398.7, and 531.0 eV, respectively. The C 1s spectrum was well deconvoluted into C=C (284.4 eV), C–C/C–P (285.0 eV), C–N (285.7 eV), C–O (286.2 eV), and O–C=O (287.8 eV) (Figure 1d). The three distinct peaks at 400.6, 399.8, and 398.5 eV in the N 1s spectrum, respectively, corresponded to C–N, N–H, and C=N–H (Figure 1e). The high-resolution scan of P 2p decomposed into two distinct peaks at 133.8 and 133.1 eV, indicating the formation of P=O and P–O, respectively (Figure 1f). The XPS results of PNCDs were consistent with those of FT-IR, suggesting that the P and N elements were successfully doped into the final product.

### 3.2. Optical Properties of PNCDs

**Figure 2:**
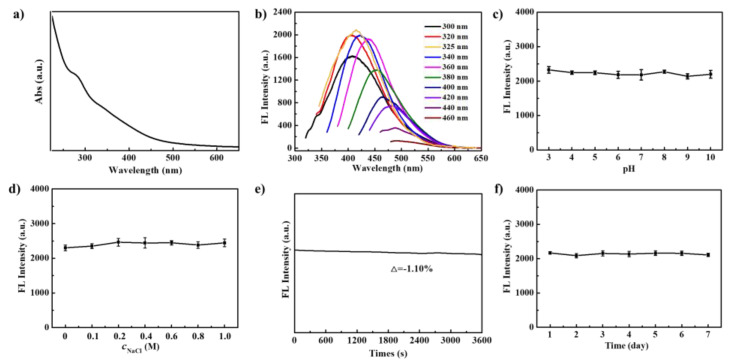
(**a**) Ultraviolet-visible (UV–vis) absorption of PNCDs, Fluorescence spectra of PNCDs at various (**b**) excitation wavelengths, (**c**) pH values, and (**d**) ionic strengths and (**e**) during 60 min of continuous irradiation and (**f**) during 7 days of storage. λex = 325 nm.

The remarkable optical features of PNCDs were explored using UV–vis and fluorescence spectroscopy. It can be seen in Figure 2a that the PNCDs exhibited intense absorption bands around 270 nm, which were attributed to the π–π* transitions of C=C/C=N/N=P groups [49]. Figure 2b shows the fluorescence behaviors of PNCDs under excitation from 300 to 460 nm. A redshift in fluorescence emission was observed, and the excitation/emission maxima were found at 325/415 nm. The CIE color parameters obtained using the PNCD emission were spectrally blue light (Figure S3). The quantum yield of PNCDs was deduced to be 22.0% using quinine sulfate (0.05 M, prepared in H_2_SO_4_) as the reference substance.

Figure 2c demonstrates that the photoluminescence of PNCDs remained quite stable at pH 3–10. Additionally, little variation in PNCD photoluminescence was observed at high ionic strengths (NaCl concentrations up to 1.0 M could be tolerated) (Figure 2d). The PL intensity of PNCDs was unchanged under 325 nm irradiation for 60 min (Figure 2e). PNCDs displayed sustained photostability after being stored at room temperature for 7 days (Figure 2f). These results suggest that PNCDs have good potential as stable fluorescent probes in biological samples where pH fluctuations, high ionic strengths, or long irradiation times might be encountered.

### 3.3. Highly Sensitive Sensing of Fe^3+^ Using PNCDs

**Figure 3:**
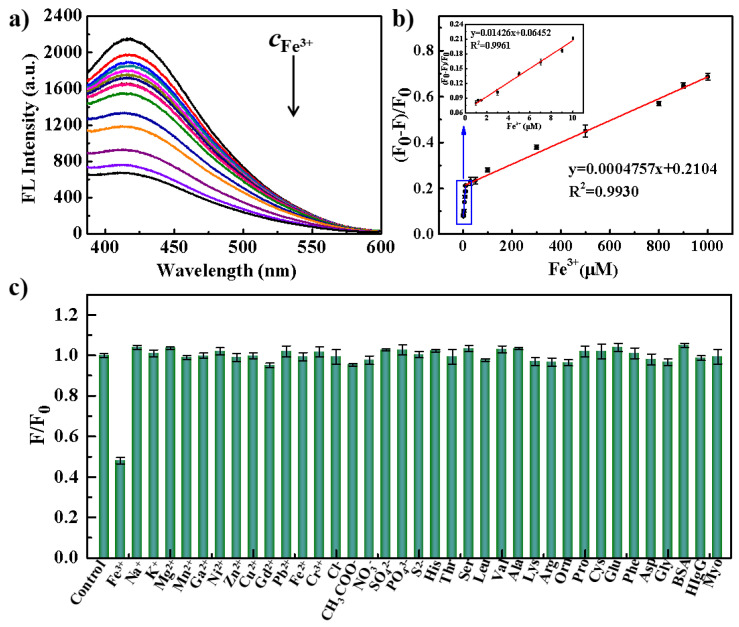
(**a**) Fluorescence responses of PNCDs in the presence of Fe^3+^ at different concentrations (0–1000 μM); (**b**) Linear relationships between fluorescence changes ((F_0_ − F)/F_0_) and Fe^3+^ concentration; (**c**) Fluorescence responses of PNCDs towards different species (500 μM ions, 100 μM amino acids, and 5 × 10^−4^ mg mL^−1^ proteins).

Inspired by the unique optical properties of the prepared PNCDs, their potential sensing applications were further investigated. An extensive screening test was performed, and distinct PL quenching of PNCDs was only observed in the presence of Fe^3+^. PL quenching was hardly influenced by pH (Figure S4), demonstrating that the PNCDs are powerful nanoprobes that can be used for Fe^3+^ sensing. It can be seen in Figure 3a that the PL intensity of PNCDs decreased with increasing Fe^3+^ concentrations. The quenching efficiency ((F_0_ − F)/F_0_) of PNCDs presents piecewise linear relationships versus the concentration of Fe^3+^ over a wide range of 1–1000 µM (Figure 3b). The linear equations are (F_0_ − F)/F_0_ = 0.01426*c*_Fe_^3+^ + 0.06452 (R^2^ = 0.9961) in the Fe^3+^ concentration range of 1–10 µM and (F_0_ − F)/F_0_ = 0.0004757*c*_Fe_^3+^ + 0.2104 (R^2^ = 0.9930) in the Fe^3+^ concentration range of 10-1000 µM. The detection limit was deduced to be 0.39 µM, which complied with the IUPAC criteria (S/N = 3). Compared to other CD probes reported for Fe^3+^ sensing, the as-prepared PNCDs had high sensing sensitivity (Table S3), which might be attributed to the favorable photostability and the effective fluorescence quenching response of PNCDs to Fe^3+^. The detection limit of the PNCDs to Fe^3+^ was far below the minimum value of the normal Fe^3+^ level in serum [50]. The as-prepared PNCDs displayed sensitive responses to a wide range of Fe^3+^. Therefore, the PNCDs could serve as powerful probes to evaluate the abnormalities of Fe^3+^ contents in bio-samples.

In order to assess the sensing selectivity of PNCDs, different ions (Na^+^, K^+^, Ca^2+^, Mg^2+^, Fe^2+^, Zn^2+^, Mn^2+^, Cu^2+^, Ni^2+^, Cd^2+^, Pb^2+^, Cr^3+^, Cl^−^, CH_3_COO^−^, NO_3_^−^, SO_4_^2−^, PO_4_^2−^, and S^2−^; 500 μM), typical amino acids (His, Thr, Ser, Leu, Val, Ala, Lys, Arg, Orn, Pro, Cys, Glu, Phe, and Leu; 100 μM) and proteins (BSA, HigG, and Myo; 5 × 10^−4^ mg mL^−1^) were used as potential interferents in the biological assay system, and the fluorescence responses of PNCDs to these species were recorded to evaluate their interference. In Figure 3c, it can be seen that significant fluorescence quenching was only observed for Fe^3+^, suggesting excellent selectivity of PNCDs toward Fe^3+^ in sensing.

### 3.4. Quenching Mechanism of PNCDs by Fe^3+^

**Figure 4:**
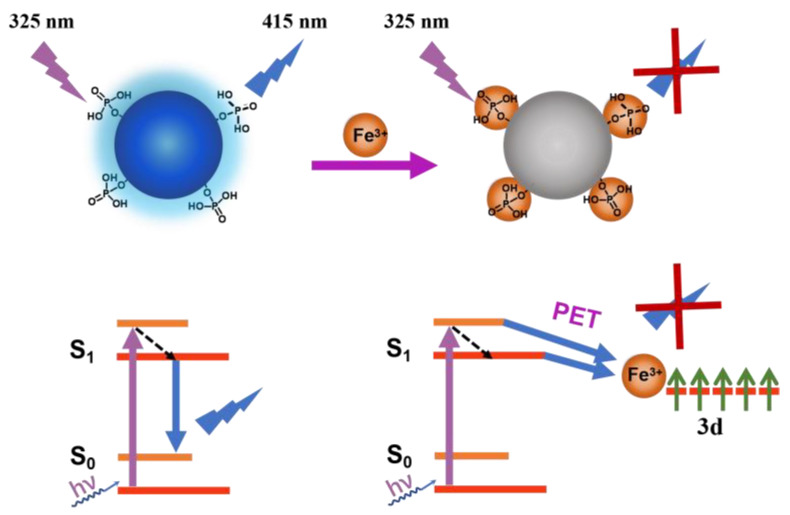
The quenching mechanism of PNCDs by Fe^3+^.

**Figure 5:**
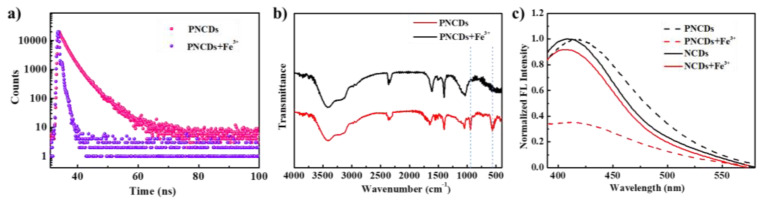
(**a**) Fluorescence lifetime decay curves of PNCDs before and after adding Fe^3+^. (**b**) FT-IR spectra of PNCDs before and after adding Fe^3+^. (**c**) Fluorescence responses of NCDs and PNCDs toward the addition of Fe^3+^.

It is well known that most transition metals are apt to form complexes owing to the existence of empty d orbitals. Fe^3+^ possesses special 3d orbits with an electron-half-filled state and can coordinate with the phosphorus groups of PNCDs to form PNCD–Fe^3+^ complexes. Compared with other ions, the electron transferred from the excited state of PNCDs to the semi-filled 3d orbitals of Fe^3+^ is easier, promoting the recombination of nonradiative electron holes and resulting in fluorescence quenching, as illustrated in Figure 4. Moreover, the high selectivity of PNCDs towards Fe^3+^ sensing may also be related to the higher thermodynamic affinity of Fe^3+^ and the faster coordination process [51]. After the chelation sites of PNCDs are occupied by Fe^3+^, other metal ions, including Fe^2+^, are not easy to combine with PNCDs, thus contributing to the favorable detection selectivity to Fe^3+^. To comprehend the PL quenching process, the fluorescence lifetimes of PNCDs with/without Fe^3+^ were determined (Figure 5a). The average lifetime of PNCDs was 5.07 ns, which decreased to 2.52 ns when adding Fe^3+^. It was indicated that a photoinduced process of electron transfer occurs between PNCDs and Fe^3+^ [52].

Figure 5b illustrates the FT-IR spectra of PNCDs before and after reaction with Fe^3+^. The bands at 562 and 1057 cm^−1^, respectively, correspond to the P–O and P=O of PNCD and disappear after the addition of Fe^3+^ due to the formation of Fe–O–P. In order to further verify the contribution of P doping to Fe^3+^ sensing, the fluorescence response of ethylenediamine-derived CDs (N-CDs) to Fe^3+^ was also recorded (Figure 5c). Compared to PNCDs, only slight quenching of the PL intensity was observed for NCDs after adding Fe^3+^, which demonstrated that the sensitive response of PNCDs towards Fe^3+^ indeed correlated closely with the doping of P in PNCDs.

### 3.5. Determination of Fe^3+^ Contents in Human Serum and SH-SY5Y Cells

The practicability of the sensitive fluorescence quench response of PNCDs was verified by determining the Fe^3+^ contents in serum and SH-SY5Y cells samples, and the determination accuracy was assessed based on the standard addition method. As depicted in Table 1, the determined values of Fe^3+^ in human serum using PNCDs as fluorescence probes were in good accordance with the normal range of Fe^3+^ levels [50], and the determined Fe^3+^ content in SH-SY5Y cell lysate agreed well with the reported value [53]. The recoveries of Fe^3+^ were in the range of 90.27–113.23%, demonstrating the reliability of the as-prepared PNCDs for application in determining the Fe^3+^ contents in biological samples.

### 3.6. Intracellular Localization of PNCDs

Favorable biocompatibility is crucial in guaranteeing the application of spectral probes in cell imaging [54]. As illustrated in Figure S5, 84% of cells remained alive when the PNCD concentration was 400 μg mL^−1^, indicating the remarkable biocompatibility of PNCDs.

**Figure 6:**
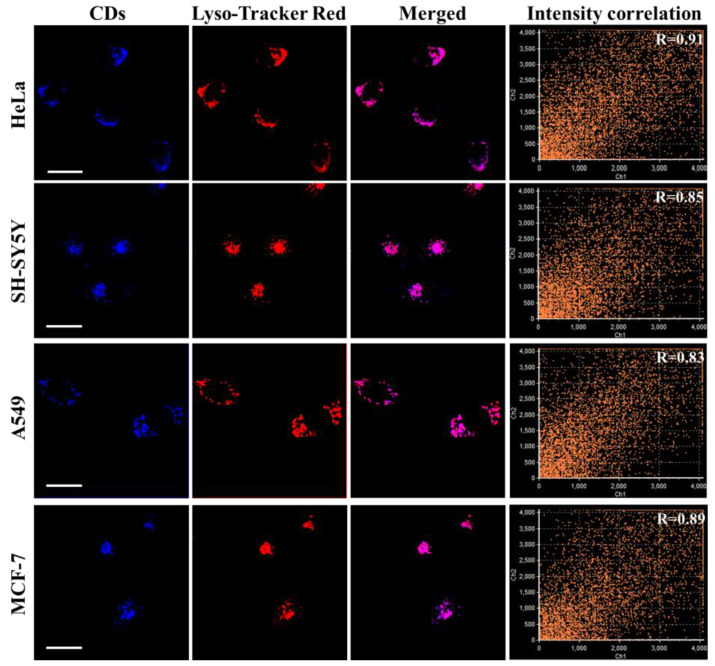
Co-localization images of PNCDs and Lyso-Tracker Red in HeLa, SH-SY5Y, A549, and MCF-7 cells.

The distributions of PNCD were investigated in different cell lines, including HeLa, SH-SY5Y, A549, and MCF-7 cells. As can be seen in Figure 6, the blue CLSM images of PNCDs overlapped well with the red CLSM images of Lyso-Tracker Red, and the Pearson’s correlation coefficients were 0.91, 0.85, 0.83, and 0.89, respectively. The results clearly indicate that the PNCDs predominantly localized to lysosomes. The excellent co-localization results indicate that the PNCDs exhibit high lysosomal location specificity and can achieve universal lysosomal staining, regardless of the cell type. On the contrary, the co-localization regions of PNCDs with ER-Tracker Red and Mito-Tracker Deep Red were significantly different (Figure S6), with low Pearson’s correlation coefficients of 0.31 and 0.63, respectively. The lysosome targeting ability was attributed to the abundance of lipophilic amino groups [55,56], which enable PNCDs to access the cells and accumulate in intracellular acidic regions due to their acidophilic effect [2,57,58]. Lysosomes are acidic organelles that can provide protons, and the protonation of the amino groups of PNCDs takes place under acidic conditions, leading to the localization of PNCDs around lysosomes following internalization [40].

**Figure 7:**
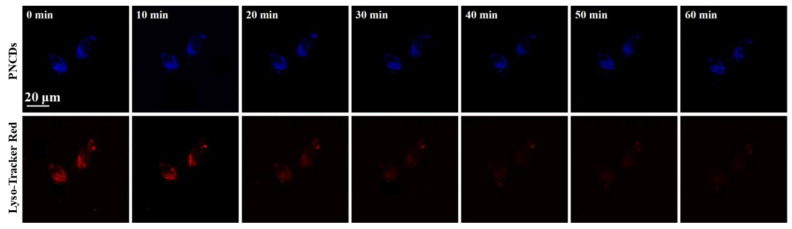
Fluorescence images of HeLa cells after staining with PNCDs and Lyso-Tracker Red, with increasing scanning times.

Figure 7 illustrates the photostability of PNCDs in cell imaging. It can be seen that the fluorescence of PNCDs remained unchanged within 60 min, while obvious fading of Lyso-Tracker Red fluorescence was observed at imaging times longer than 20 min. The excellent anti-photobleaching property of PNCDs makes them promising probes for the long-term monitoring and tracking of lysosomes.

### 3.7. Lysosomal Tracking in Cells with PNCDs

**Figure 8:**
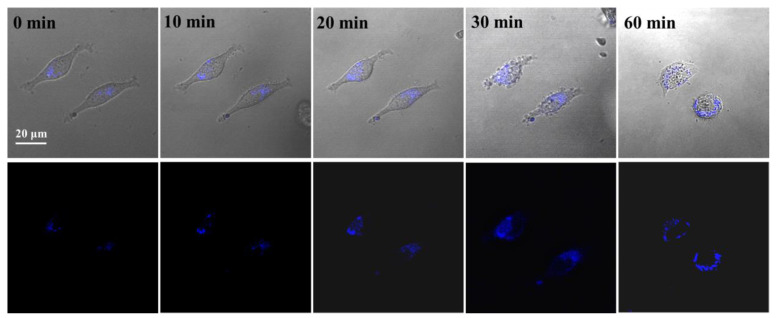
Snapshots of lysosomes in HeLa cells undergoing apoptotic cell death.

It has been reported that apoptosis can affect the function of lysosomes, and the increase in total lysosome volume is a common feature of apoptotic processes [59,60]. Dexamethasone is an anti-inflammatory agent that can induce cellular apoptosis due to proton leakage [35]. Figure 8 shows the results from the imaging of HeLa cells after a dexamethasone treatment. It can be clearly observed that the cell shape changed from spindle (in normal healthy cells) to circle (in affected cells). Meanwhile, the fluorescence images indicate that the volumes of lysosomes were significantly enlarged to the point of rupture during the cellular apoptotic death process, which was accompanied by changes in fluorescence intensity and location. The successful real-time tracking of lysosome morphology in HeLa cells sufficiently demonstrated the great potential of the as-prepared PNCDs for application as powerful probes in cytology.

## 4. Conclusions

In summary, fluorescent P and N co-doped CDs were facilely fabricated by a hydrothermal treatment of phytic acid and ethylenediamine. The introduced phosphorus groups resulted in the production of sensitive probes for selective Fe^3+^ sensing in serum samples. The formed lipophilic amino groups offer favorable lysosome location specificity, and the excellent photo-/pH stability means the prepared PNCDs are suitable for long-term lysosomal tracking in living cells, which might provide useful information regarding lysosome-related diseases and in-clinic diagnosis. The method for the fabrication of the proposed CDs represents not only a means for providing efficient probes for sensing/imaging but also a practical strategy for regulating the physicochemical features and optical properties of CDs through the rational selection of precursors for multifunctional applications.

## Figures and Tables

**Table 1:** Determination of Fe^3+^ contents in human serum samples (n = 3).

Sample	Determined (μM)	Added (μM)	Found (μM)	Recovery (%)	RSD (%, n = 11)
Serum 1	2.93 ± 0.23	5	7.44 ± 0.17	90.27 ± 1.70	1.31
Serum 2	3.22 ± 0.86	5	8.88 ± 0.78	113.23 ± 1.77	1.26
Serum 3	3.86 ± 0.05	5	8.65 ± 0.03	95.76 ± 0.47	0.94
Serum 4	4.04 ± 0.39	5	9.58 ± 0.22	110.33 ± 3.58	1.74
SH-SY5Y cells	2.58 ± 0.25	5	7.70 ± 0.31	102.33 ± 6.07	1.42

## Data Availability

The data presented in this work are available in the article.

## Supplementary Materials

The following supporting information can be downloaded at https://www.mdpi.com/article/10.3390/bios13020230/s1. Figure S1: (a) TEM image, (b) XRD pattern, (c) FT-IR spectrum, (d) XPS spectrum (e) UV–vis absorption, and (f) fluorescence spectra of NCDs; Figure S2: XRD pattern of PNCDs; Figure S3: CIE 1931 coordinates of PNCDs (black dot); Figure S4: Normalized PL intensity of PNCDs before and after adding Fe^3+^ at 415 nm under different pH conditions; Figure S5: Cytotoxicity of PNCDs; Figure S6: Fluorescence images of HeLa cells co-cultured with PNCDs and other organelle probes (ER-Tracker Red and Mito-Tracker Deep Red); Table S1: Summary of the preparation and applications of N and P co-doped CDs; Table S2: Quantum yields of PNCDs with different phytic acid/ethylenediamine mole ratios; Table S3: Performance of CD-based fluorescence probes in Fe^3+^ sensing [52,61,62,63,64,65,66,67,68,69,70,71,72,73,74,75].
