# Supplementary material for: Fabrication of P and N Co-Doped Carbon Dots for Fe3+ Detection in Serum and Lysosomal Tracking in Living Cells
Source: Biosensors (Basel). 2023 Feb 5;13(2):230. doi: 10.3390/bios13020230 (PMC9954533; doi:10.3390/bios13020230)
Supplement: Supplementary file 1 [file biosensors-13-00230-s001.zip › biosensors-2131135-supplementary.pdf]

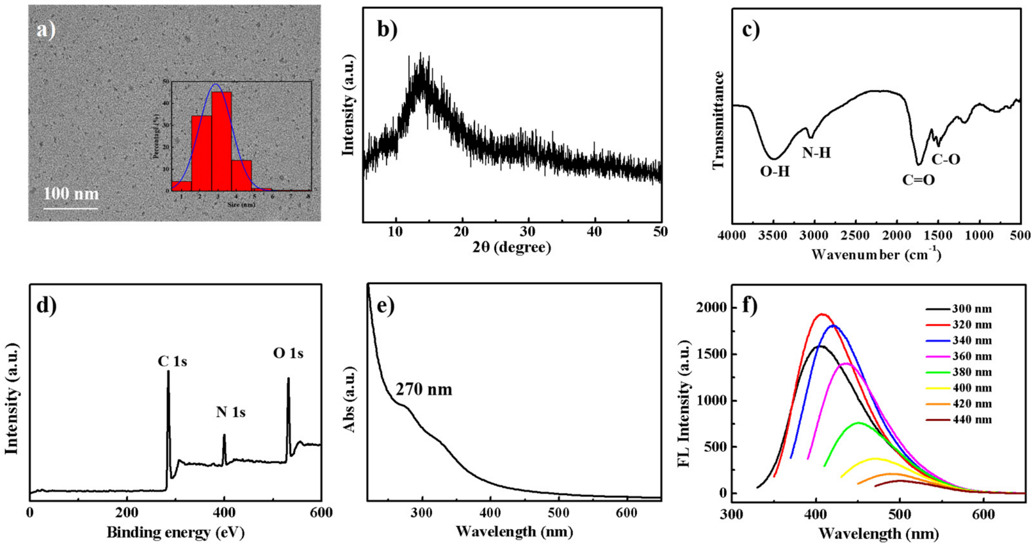

Figure S1. (a) Transmission electron microscopy (TEM) image, (b) X-ray powder diffra ction (XRD) pattern, (c) Fourier transform infrared spectrum (FT-IR), (d) X-ray photoelectron spectroscopy (XPS) spectrum (e) Ultraviolet-visible (UV–vis) absorption, and (f) Fluorescence spectra of NCDs.

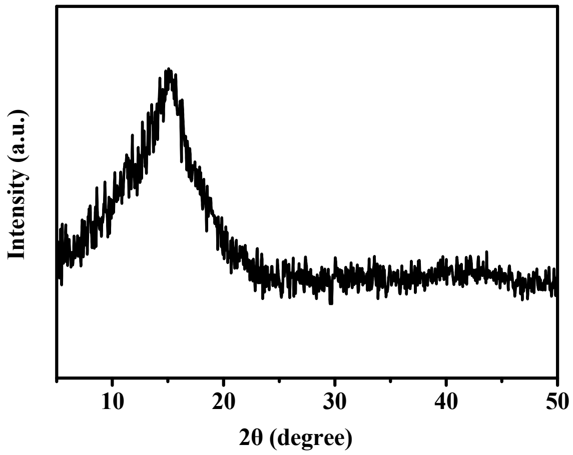

Figure S2. XRD pattern of PNCDs.

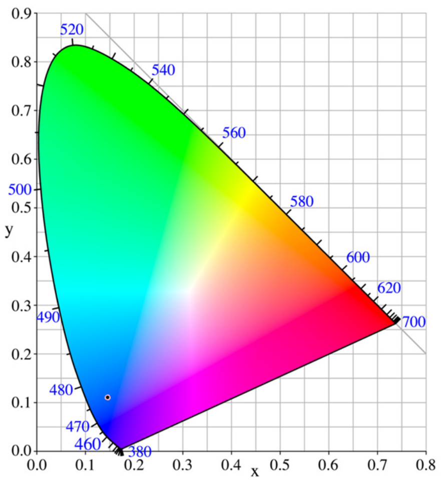

Figure S3. CIE 1931 coordinates of PNCDs (black dot).

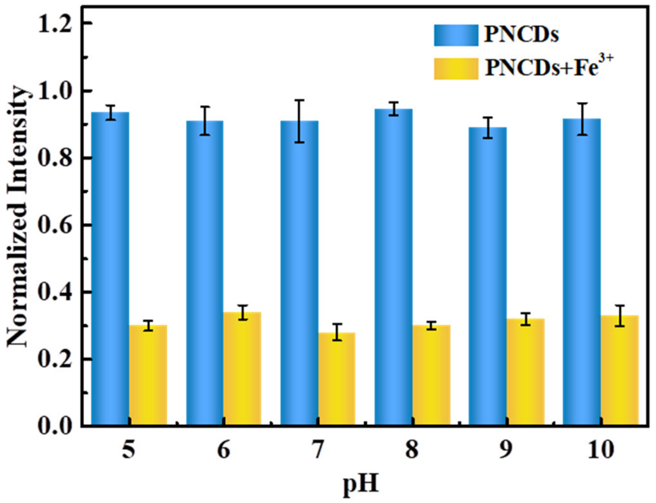

Figure S4. Normalized PL intensity of PNCDs before and after adding Fe^{3+} at 415 nm under different pH conditions.

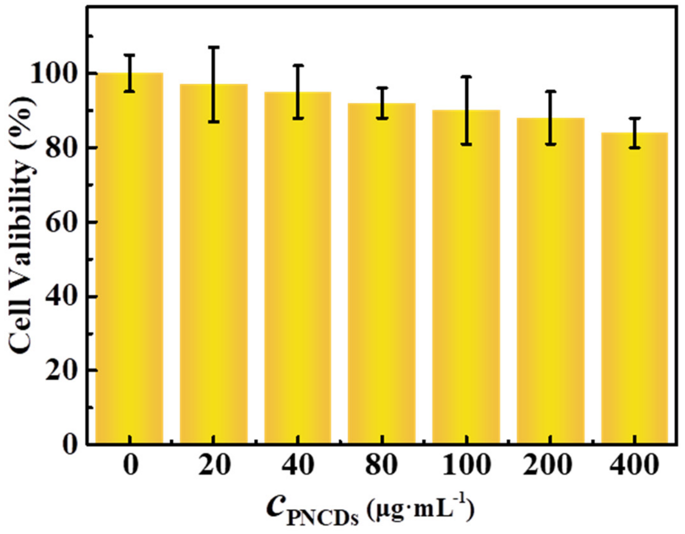

Figure S5. Cytotoxicity of PNCDs.

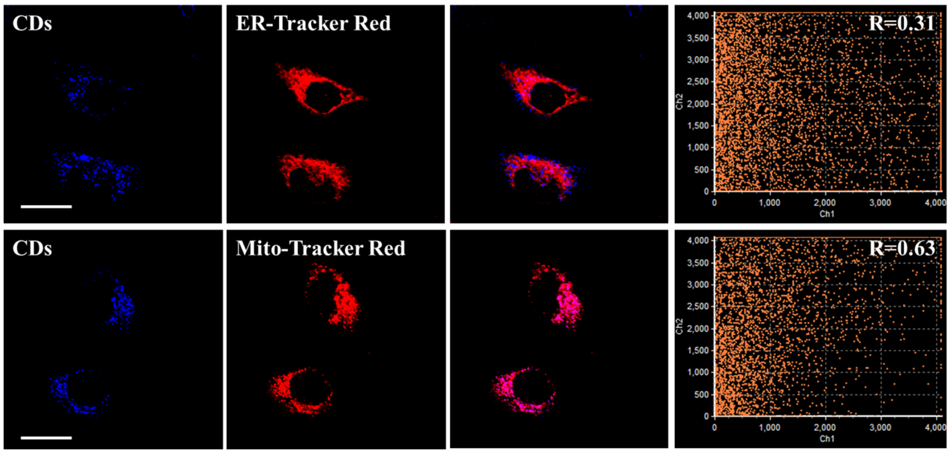

Figure S6. Fluorescene images of HeLa cells co-cultured with PNCDs and other organelle probes (ER-Tracker Red and Mito-Tracker Red).

Table S1. Summary on the preparation and applications of N,P co-doped CDs

Precursors	Method of preparation	Applications	Ref
Ascorbic acid, polyethylenimine and phosphoric acid	Carbonization	NO_2^- Detection	[61]
Sucrose, phosphoric acid and 1,2-ethylene-diamine	Carbonization	Vitamin B_{12} and Co^{2+} Detection	[62]
D-glucose and L-aspartic acid	Pyrolysis	Brain cancer cells Diagnosis	[63]
Glucose, phosphoric acid and polyeth-ylene glycol diamine	Carbonization	Cu^{2+} Detection	[64]
Citric acid, ethylenediamine and urea phosphate	Microwave digestion	Dopamine Detection	[65]
Ophosphorylethanolamine and citric acid.	Hydrothermal	Cd^{2+} Detection	[66]
1,4-naphthalenedi-carboxylic acid, urea and H_3PO_4	Solvothermal	Microbial viability Assessment	[67]

Table S2. Quantum yields of PNCDs under different phytic acid/ethanediamine mole ratios.

Phytic acid/ ethylenediamine mole ratio	Reaction temperature (℃)	Reaction time (h)	Quantum yield (%)
10:3	200	4	0.8
5:3	200	4	0.2
1:3	200	4	1.9
1:15	200	4	14.8
1:21	200	4	22.0
1:30	200	4	12.1

Table S3. Performance of CDs-based fluorescence probes for sensing Fe^{3+}.

CDs	Linear range (μM)	LOD (μM)	Ref.
NCDs	0-50	0.8	[68]
NFCDs	5-30	1.03	[69]
NSCDs	-	0.854	[52]
NSCDs	40-600	0.45	[70]
NSCDs	25-500	4	[71]
CDs	8-80	3.8	[72]
NCDs	0-70	0.50	[73]
Phe-CDs	5-500	0.72	[74]
CDs	33-133	0.53	[75]
PNCDs	1-1000	0.39	This work
